# Formation of precise 2D Au particle arrays via thermally induced dewetting on pre-patterned substrates

**DOI:** 10.3762/bjnano.2.37

**Published:** 2011-06-22

**Authors:** Dong Wang, Ran Ji, Peter Schaaf

**Affiliations:** 1 Department of Materials for Electronics, Institute of Materials Engineering and Institute of Micro- and Nanotechnologies MacroNano®, Ilmenau University of Technology, POB 10 05 65, 98684 Ilmenau, Germany; 2 SUSS MicroTec Lithography GmbH, Schleissheimer Str. 90, 85748 Garching, Germany

**Keywords:** Au particles, dewetting, nanoimprint lithography, nanoparticle array

## Abstract

The fabrication of precise 2D Au nanoparticle arrays over a large area is presented. The technique was based on pre-patterning of the substrate before the deposition of a thin Au film, and the creation of periodic particle arrays by subsequent dewetting induced by annealing. Two types of pre-patterned substrates were used: The first comprised an array of pyramidal pits and the second an array of circular holes. For the dewetting of Au films on the pyramidal pit substrate, the structural curvature-driven diffusion cooperates with capillarity-driven diffusion, resulting in the formation of precise 2D particle arrays for films within a structure dependent thickness-window. For the dewetting of Au films on the circular hole substrate, the periodic discontinuities in the films, induced by the deposition, can limit the diffusion paths and lead to the formation of one particle per individual separated region (holes or mesas between holes), and thus, result in the evolution of precise 2D particle arrays. The influence of the pre-patterned structures and the film thickness is analyzed and discussed. For both types of pre-patterned substrate, the Au film thickness had to be adjusted in a certain thickness-window in order to achieve the precise 2D particle arrays.

## Introduction

An increasing amount of scientific attention is being paid to the ordered arrangement of metallic nanoparticles, due to their wide range of potential applications in plasmonics [1–2], magnetic memories [3], DNA detection [4], and catalysis for nanowire and nanofiber growth [5–6]. Nanoparticle arrays are typically fabricated either by chemical processes based on self-assembly or by lithography based nanostructuring. The synthesis of 3D arrays of ligand stabilized Au nanoparticles using the self-assembly method has been reported [7], and electron beam lithography has also been used to define positioned nanoparticles at low throughput [8]. Another simple method for the formation of nanoparticle arrays is based on the dewetting process of thin metal films on an inert substrate. Dewetting of metallic films on a substrate is driven by the reduction of the surface energy of the thin film and of the interface energy between the film and substrate, and can be induced by thermal annealing [9–11], pulsed laser heating [12–20], ion irradiation [21–24], and electron irradiation [25]. Dewetting proceeds by surface diffusion even in the solid state well below the melting temperature of the film [9–11]. In addition, metals such as Ni, Ag, Co, and Au have a weak interaction with SiO_2_ substrate, which results in low activation energy for metal atom migration [12].

Dewetting is a well known spontaneous physical phenomenon describing the rupture of a thin liquid film on a substrate and the formation of droplets. Dewetting dynamics of liquid polymer films have been studied [26–29] and three dewetting mechanisms are known: (1) Heterogeneous nucleation, which initiates from a defect located at the film surface or the film–substrate interface, (2) homogeneous nucleation, which occurs via a small thermal density fluctuation that acts as a nucleus for hole formation, and (3) spinodal dewetting, which occurs by the amplification of periodic film thickness fluctuations (i.e., capillary wave); such films induce self-correlated dewetting patterns [25].

Recently, dewetting of solid films has also been studied. Theoretically, for the defect-free and homogenous films, the surface energy driven mechanism starts at the film boundary, with edge agglomeration via capillary edge instability, and is then followed by particle formation via Rayleigh instability [30–31]. However, real films comprise defects and fluctuations in the film thickness. As well as by edge agglomeration [32–33], voids can nucleate due to periodic film thickness fluctuations (spinodal dewetting), or at defects, which is then followed by void growth and particle formation [31]. For polycrystalline metallic films, dewetting is also affected by the character of grain boundaries [33]. Altogether, dewetting of polycrystalline metallic films on a flat substrate usually leads to a broad distribution of particle size and spacing.

On the other hand, dewetting of metallic films on pre-patterned substrates can lead to the formation of ordered particle arrays. Formation of 2D ordered arrays of nanoparticles was observed on a thin metal film that had been patterned using focused ion beam (FIB) before the dewetting process [34]. However, the FIB patterning is a time-consuming process. Giermann and Thompson reported the formation of a 2D ordered Au nanoparticle array, with uniform size and aligned crystallographic orientation, on a substrate with an array of periodic pits, via solid-state dewetting induced by annealing at 850 °C [9]. Our previous work showed that a pre-patterned substrate with deep grid grooves can also lead to the formation of a 2D ordered Au nanoparticle array via dewetting induced by annealing at 900 °C [11]. Elsewhere, a 2D ordered Au nanoparticle array was formed on a nano-hole patterned substrate via electron-beam-induced dewetting of the Au thin film [25]. Ripple patterned SiO_2_ substrates [35] and stepped alumina substrates [36] also led to the formation of metallic nanoparticle arrays via thermal dewetting. The explanation for this is that the structural curvature and the corresponding chemical potential is modulated by the pre-patterned substrate structure, and thereby the ordered particle array is prone to evolve [9,11,25,35–36]. Here, thermal dewetting of the Au films. induced by annealing, has been studied, on both the flat substrate and two types of pre-patterned substrates (one with an array of pyramidal pits and one with an array of circular holes, made using nanoimprint lithography), and large areas of 2D ordered nanoparticle arrays were fabricated. Instead of the mechanism based on the modulation of chemical potential, as presented in the previous work, it is found here that the deposition-induced periodic discontinuities of the Au films, on the substrate with an array of circular holes, limited the diffusion path and resulted in the formation of 2D ordered nanoparticle arrays with well defined particle size and spacing more effectively.

## Results

**Figure 1:**
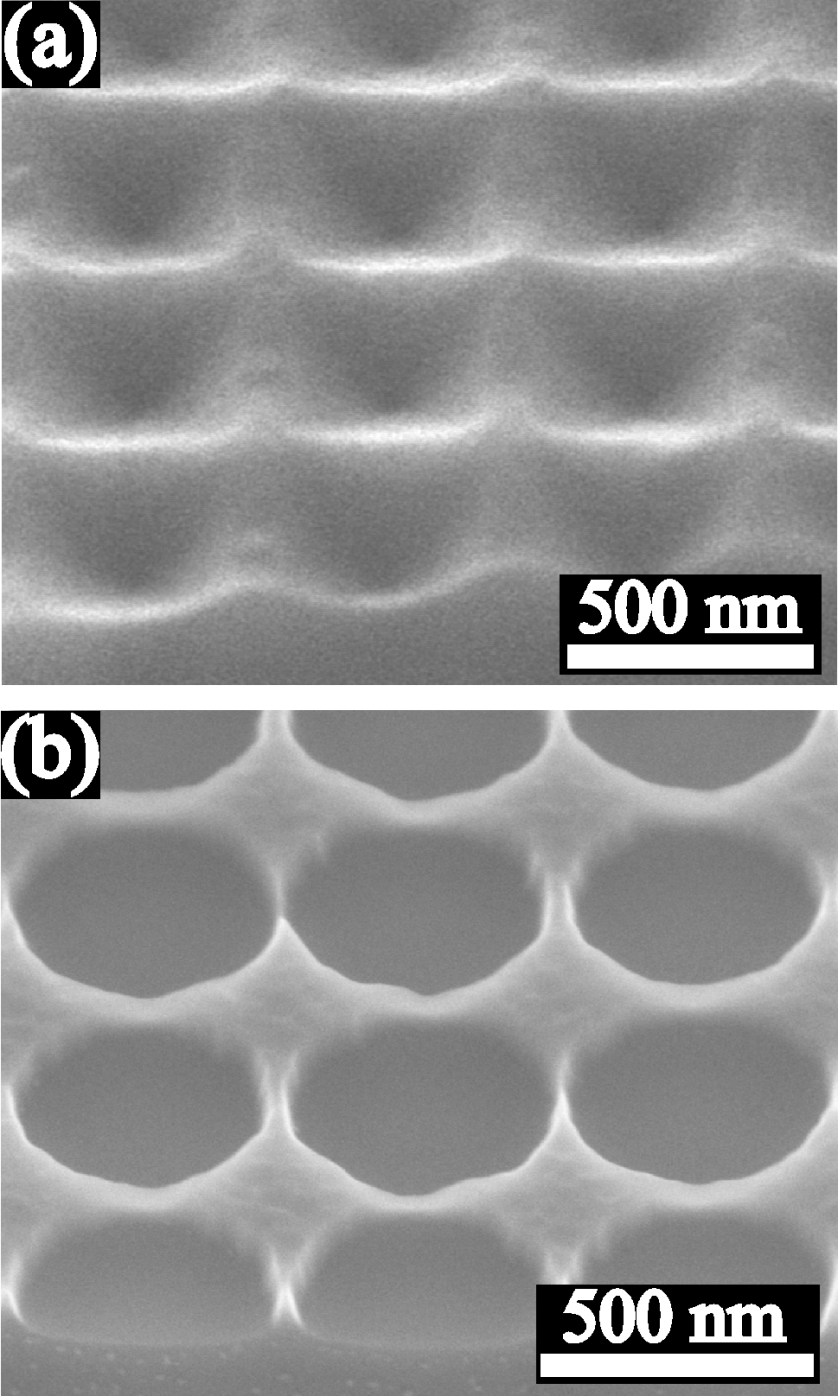
SEM images at 45° tilt of (a) a square array of pyramidal pits (substrate A) and (b) an array of circular holes with square symmetry (substrate B).

Au films were deposited on flat, and two types of pre-patterned, SiO_2_/Si substrates for the thermal dewetting induced by an annealing process at 900 °C in N_2_. Both types of pre-patterned substrates were fabricated using substrate conformal imprint lithography (SCIL) [37] and investigated by scanning electron microscopy (SEM): One with a square array of pyramidal pits (substrate type A), shown in Figure 1a, and another with an array of circular holes with square symmetry (substrate type B), shown in Figure 1b. The pits in substrate A have a spatial period of 513 nm and a depth of 150 nm. The holes in the substrate B have the same spatial period of 513 nm, a diameter of about 490 nm, and a depth of 120 nm.

**Figure 2:**
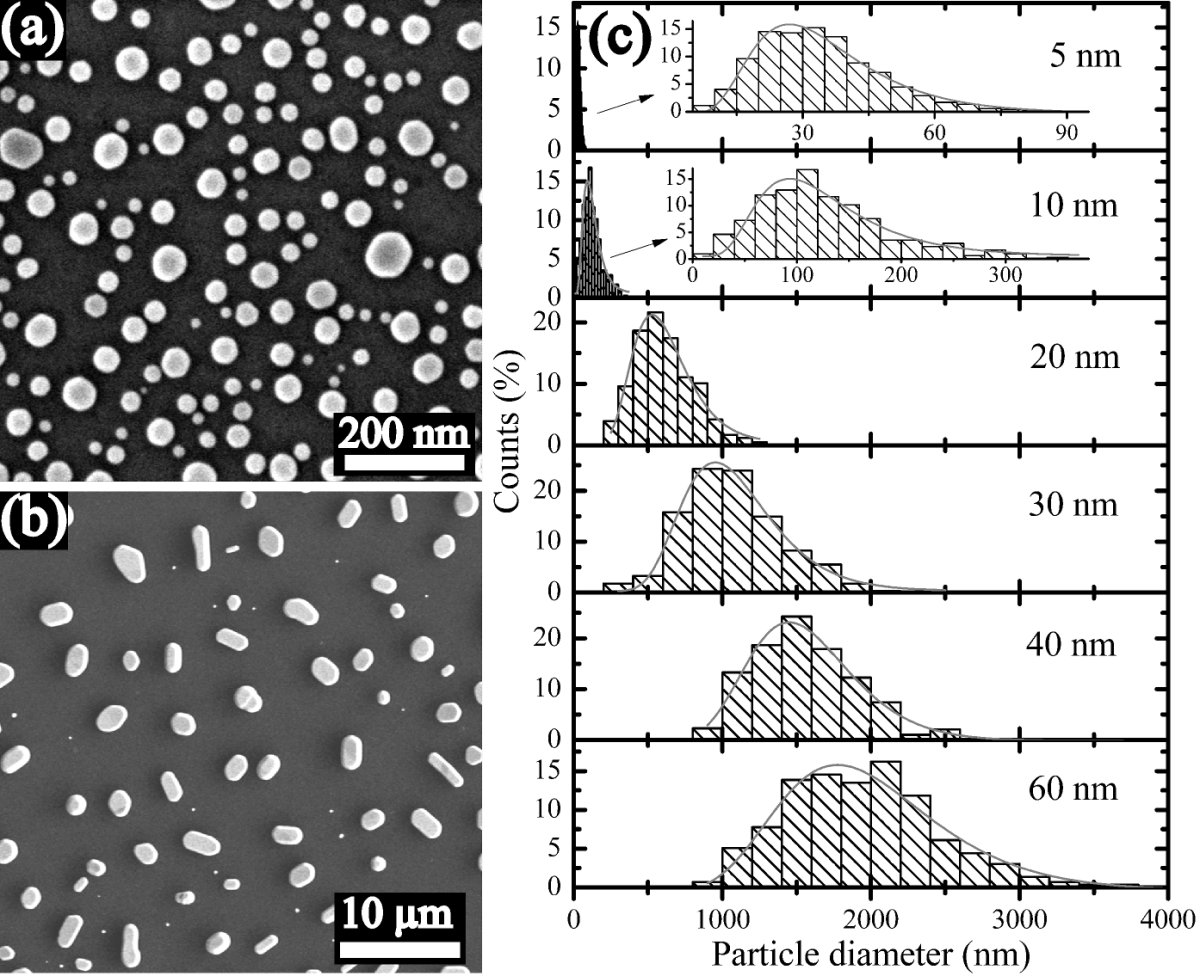
SEM images of induced particles on the flat SiO_2_/Si substrate after dewetting of the 5 nm (a) and 60 nm (b) thick Au films. (c) Histograms of particle size distributions produced by the dewetting of the 5 nm, 10 nm, 20 nm, 30 nm, 40 nm, and 60 nm thick Au films on the flat SiO_2_/Si substrates. Inset plots in (c) are magnified for clarity. Fitting curves (log-normal function) are superimposed on the histograms.

Figure 2 shows the SEM images of the Au particles formed from the 5 nm and 60 nm thick Au films on a flat SiO_2_/Si substrate. Usually, flat substrates lead to a broad distribution of particle size and spacing of the dewetted particles. Figure 2c shows the particle size distributions produced by dewetting of Au films with thicknesses from 5 nm to 60 nm on the flat substrates. Both, mean particle size <*d*_p_> and the width of particle size distribution σ_p_ increase with increasing film thickness.

**Figure 3:**
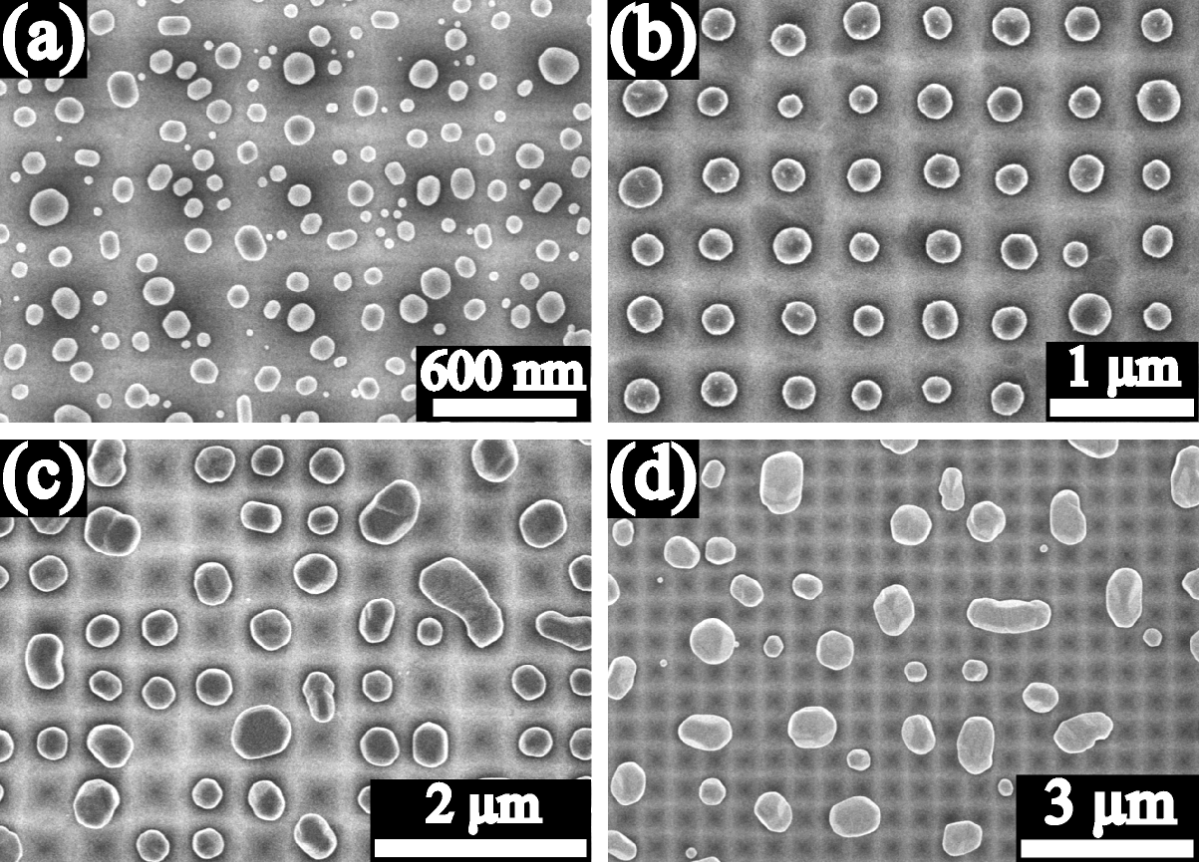
SEM images of Au particles produced on the 10 nm (a), 20 nm (b), 40 nm (c), and 60 nm (d) thick Au films on substrate A (pyramidal pits).

Figure 3 shows the SEM images of the Au particles produced from the 10 nm, 20 nm, 40 nm, and 60 nm thick Au films on the substrate A (pyramidal pits). For the 10 nm thick film, several particles could be observed in any one pit. Furthermore, there were relatively more larger size particles located in the pits than on the ridges between pits, as seen in Figure 3a. For the 20 nm thick film, all particles were located in the pits and in every pit there was only one particle. Thus a periodic array of particles evolved as seen in Figure 3b. For the 40 nm thick film, some pits are empty, while the size of some particles is clearly larger than the spatial period of the pyramidal pits and sometimes even two pits were occupied by one large particle, as seen in Figure 3c. For the 60 nm thick film, many more pits are empty and more large individual particles occupying two or more pits are formed, as seen in Figure 3d.

**Figure 4:**
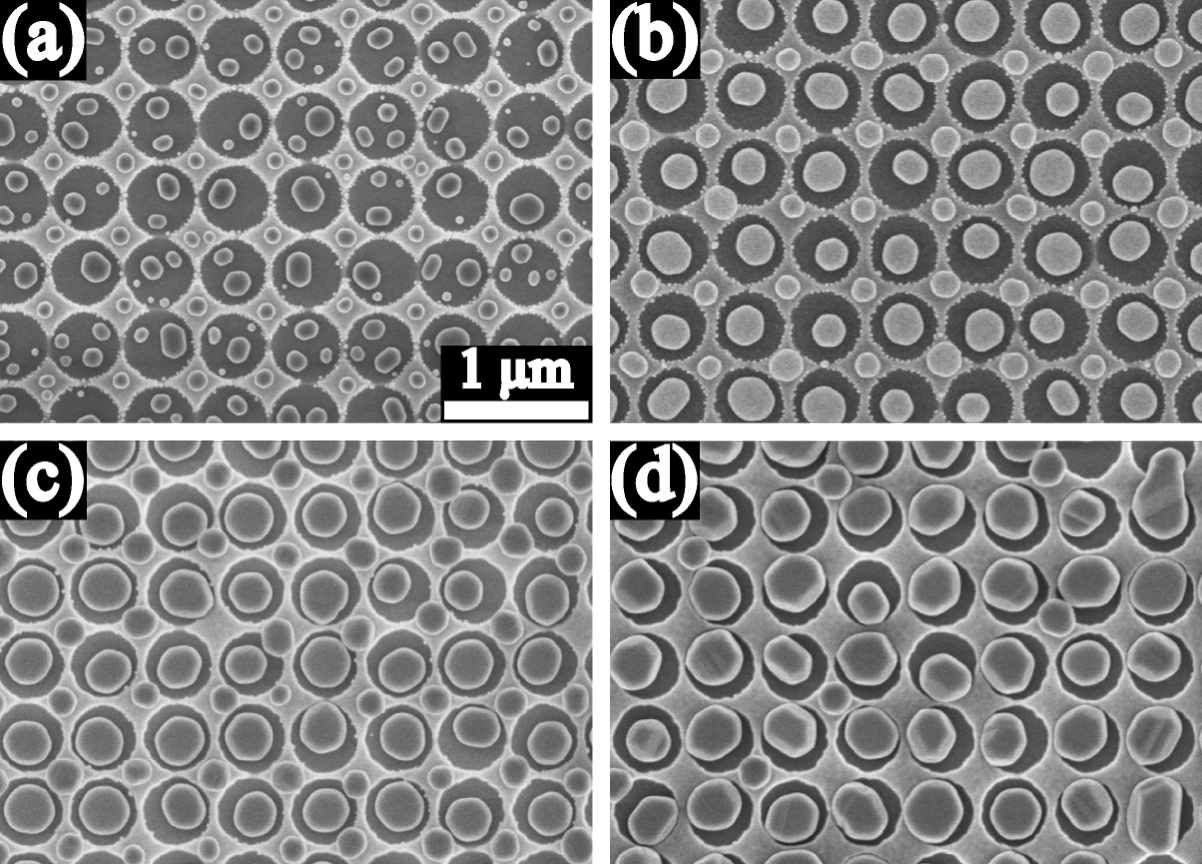
SEM images of Au particles produced from the 10 nm (a), 20 nm (b), 40 nm (c), and 60 nm (d) thick Au films on substrate B (circular holes). The scale bar 1 µm is valid for all 4 images.

The influence of substrate B (circular holes) on the dewetting was somewhat different. Figure 4 shows the SEM images of the dewetted Au particles on the substrate B, which were formed from the 10 nm, 20 nm, 40 nm, and 60 nm thick Au films. For the 10 nm thick film, several particles were often observed in one individual hole, and for the most part, only one particle with similar size was located on each of the mesas between holes (Figure 4a). Further SEM investigation showed that the 10 nm thick as-deposited film contained a number of substrate-exposing grooves. These grooves play an important role for the dewetting process. Au films retract from the edge of the grooves and the grooves expand, such that multiple particles are formed per pit (Figure 3a) or per hole (Figure 4a). For the 20 nm thick Au film, there was only one particle formed in every hole and one on every mesa (Figure 4b). The particles in the holes are clearly larger than the particles on the mesas. In addition, particle chains consisting of much smaller particles were formed on the circular interior walls of the holes for the cases of the 10 nm and 20 nm thick Au films (Figure 4a and b). For the 40 nm thick Au film, similarly, only one particle was formed in every hole and on every mesa (Figure 4c), but particle chains around the interior walls of the holes were not observed. For the case of the 60 nm thick Au film, most individual holes were filled with one particle, but on most mesas there was no particle located (Figure 4d).

**Figure 5:**
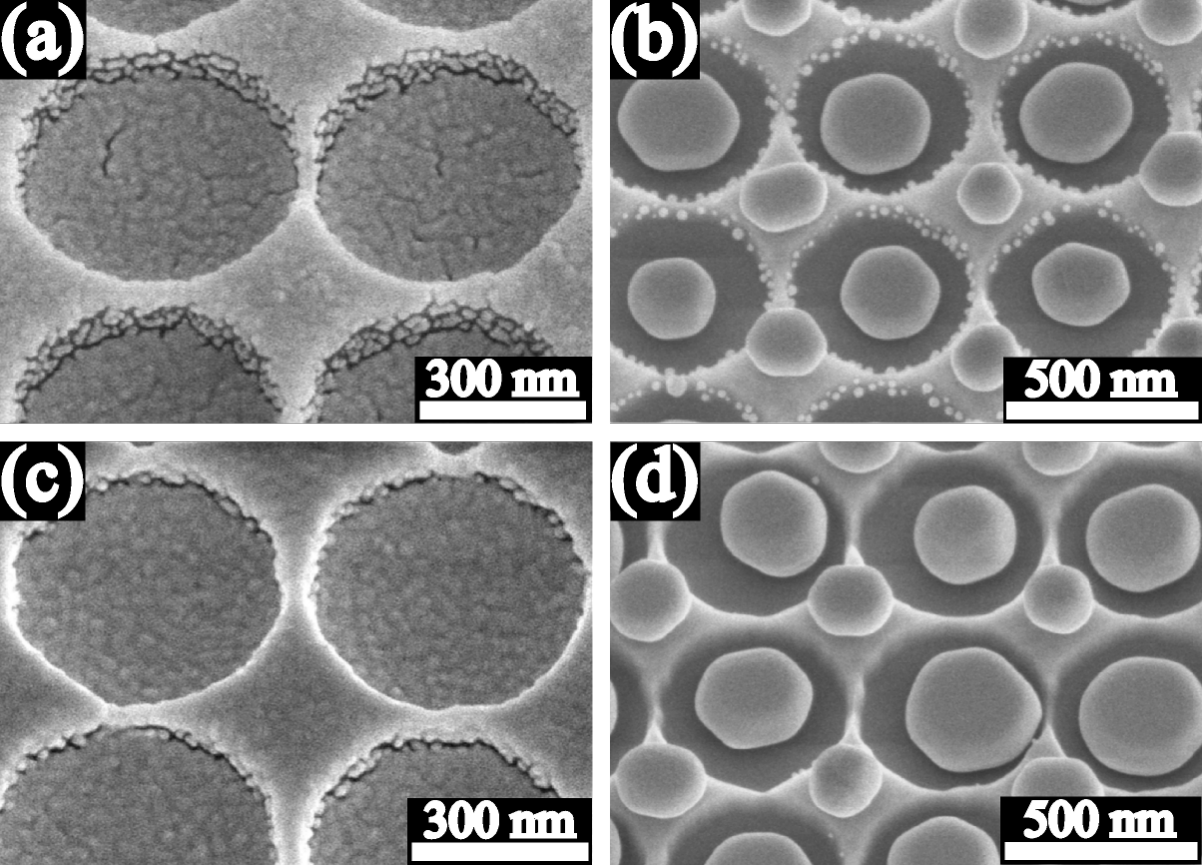
SEM images at 30° tilt of the 20 nm (a) and 40 nm (c) thick as-deposited Au films, and the corresponding particles (b) and (d) after dewetting on substrate B (circular holes).

Figure 5 shows the magnified SEM images at 30° tilt of the 20 nm and 40 nm thick as-deposited Au films, and the corresponding dewetted particles on the substrate B. On the interior walls of the holes, discontinuity can be observed in both the 20 nm and 40 nm thick as-deposited Au films (Figure 5a and c). The discontinuity morphology on the interior walls also changes with film thickness. When the film is initially very thin, the discontinuity region consists of isolated islands, for example in the 10 nm and 20 nm thick as-deposited films (Figure 5a). During dewetting, the isolated islands retract into small particles and thereby the chains of small particles can evolve (Figure 5b). As the thickness increases, the discontinuity region changes in form and consists of substrate-exposing grooves (Figure 5c). The films retract from the groove edges, such that no small particles remain on the interior walls after dewetting (Figure 5d). The mean width of grooves decreases with increasing film thickness. Finally, if the thickness was increased further, the discontinuity on the interior walls should disappear.

**Figure 6:**
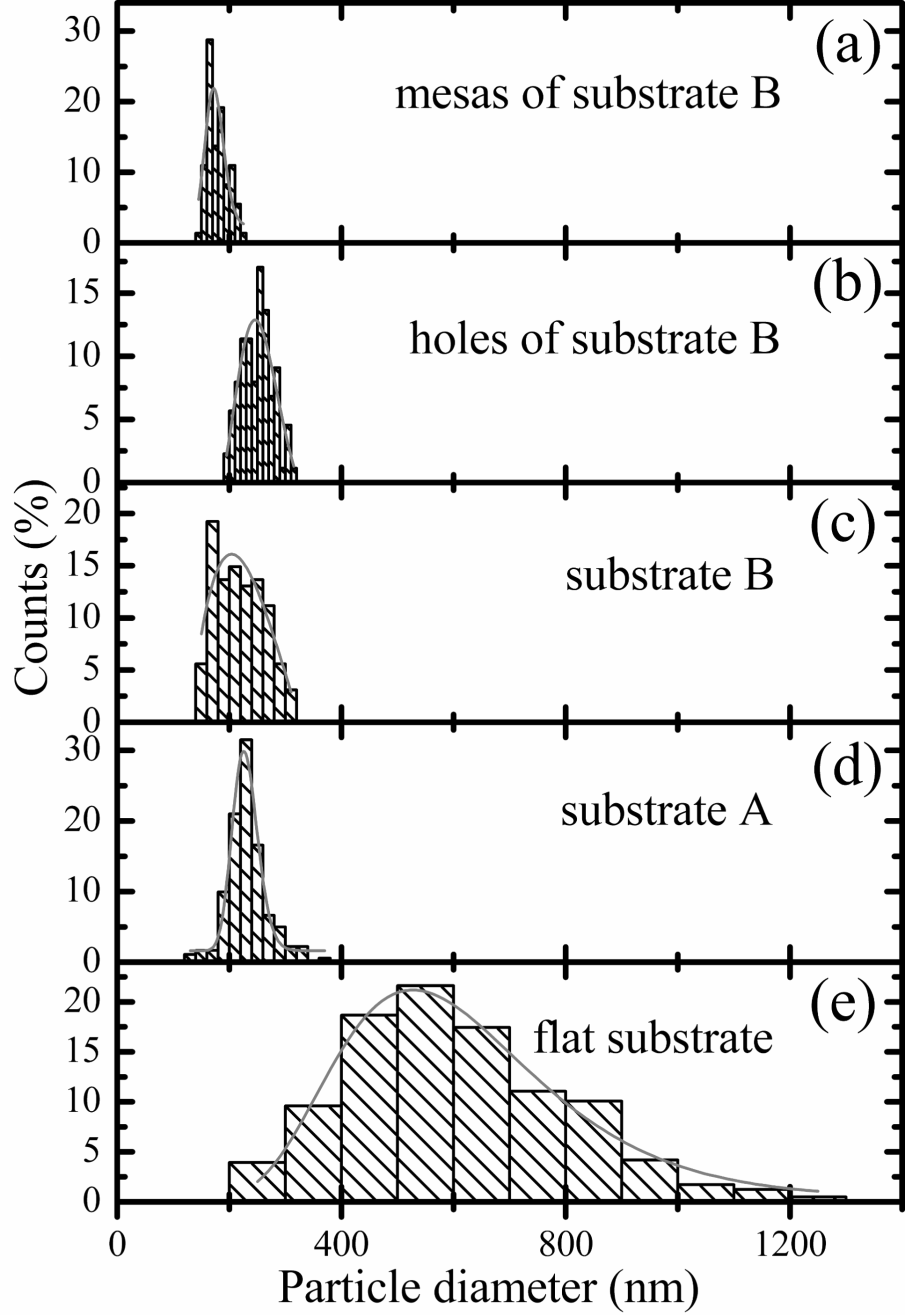
Histograms of particle size distributions, produced by dewetting of the 20 nm thick Au films, on the mesas of the substrate B (a), in the holes of the substrate B (b), on the substrate B (c), on the substrate A (d), and on the flat substrate (e). Fitting curves (fitted with log-normal function) are superimposed on the histograms.

Figure 6 shows the particle size distributions produced by dewetting of the 20 nm thick Au films on the different substrates. Comparing to the flat substrate (Figure 6e), both pre-patterned substrates A and B (Figure 6c and d) led to a clear reduction of absolute particle size and the width of the particle size distribution, i.e., the particle size is became smaller and more uniform. For a given film thickness, the particle size for the evolved particle arrays on both substrate A and substrate B is related to the individual structure dimension. Substrate A results in particle size distributions with clearly sharper peaks than substrate B, on which dewetted particles locate in two different regions: In the holes and on the mesas. Particle size distributions for particles in the holes (Figure 6b) and on the mesas (Figure 6a) of the substrate B are also plotted for comparison, and it is clear that the particles formed in holes generally are larger.

**Figure 7:**
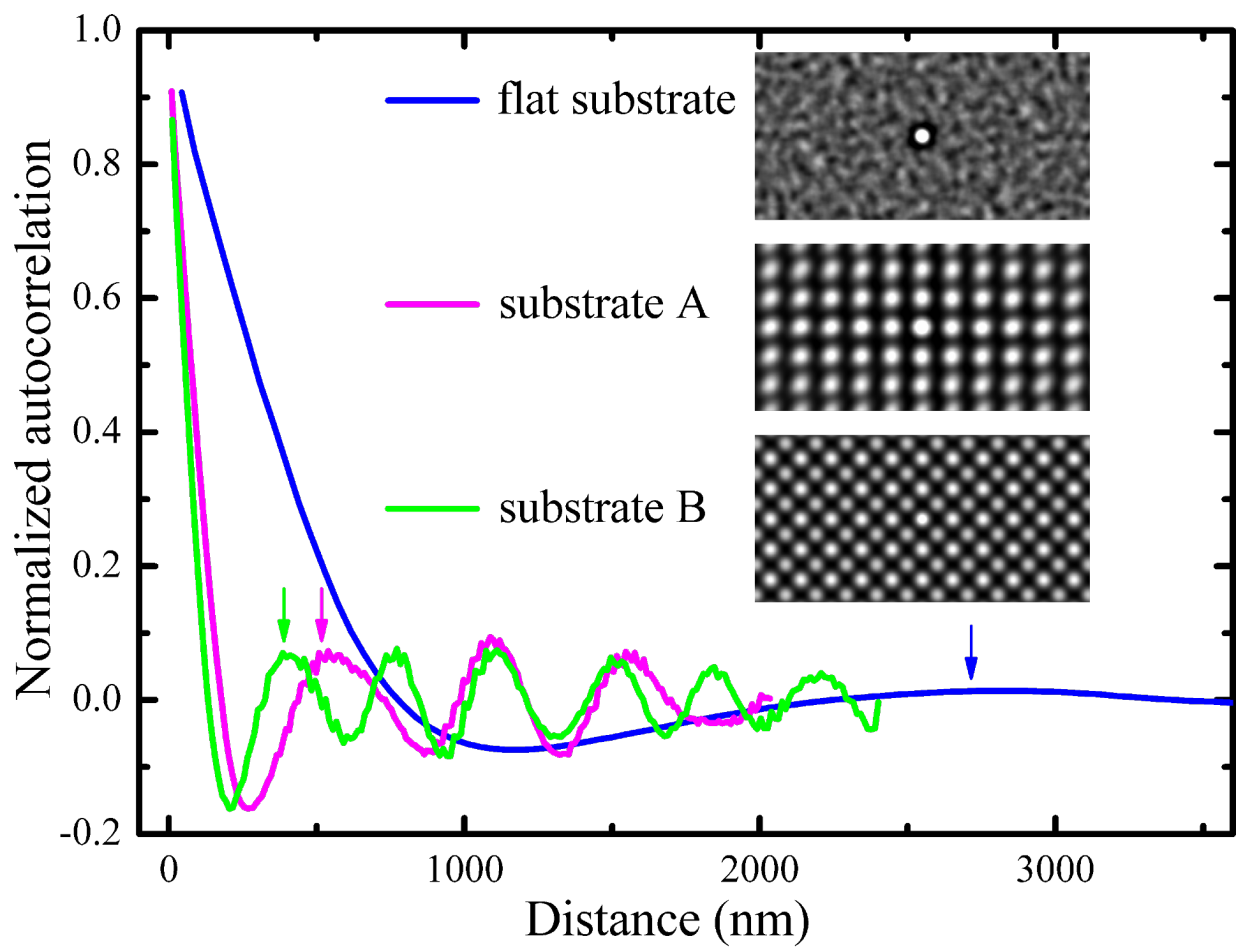
Plot of radially averaged autocorrelation of the induced particles of the 20 nm thick Au films on the flat substrate and on substrates A and B. Arrows indicate the corresponding characteristic particle spacing *s* (at maximum). Insets show the corresponding autocorrelation images.

**Figure 8:**
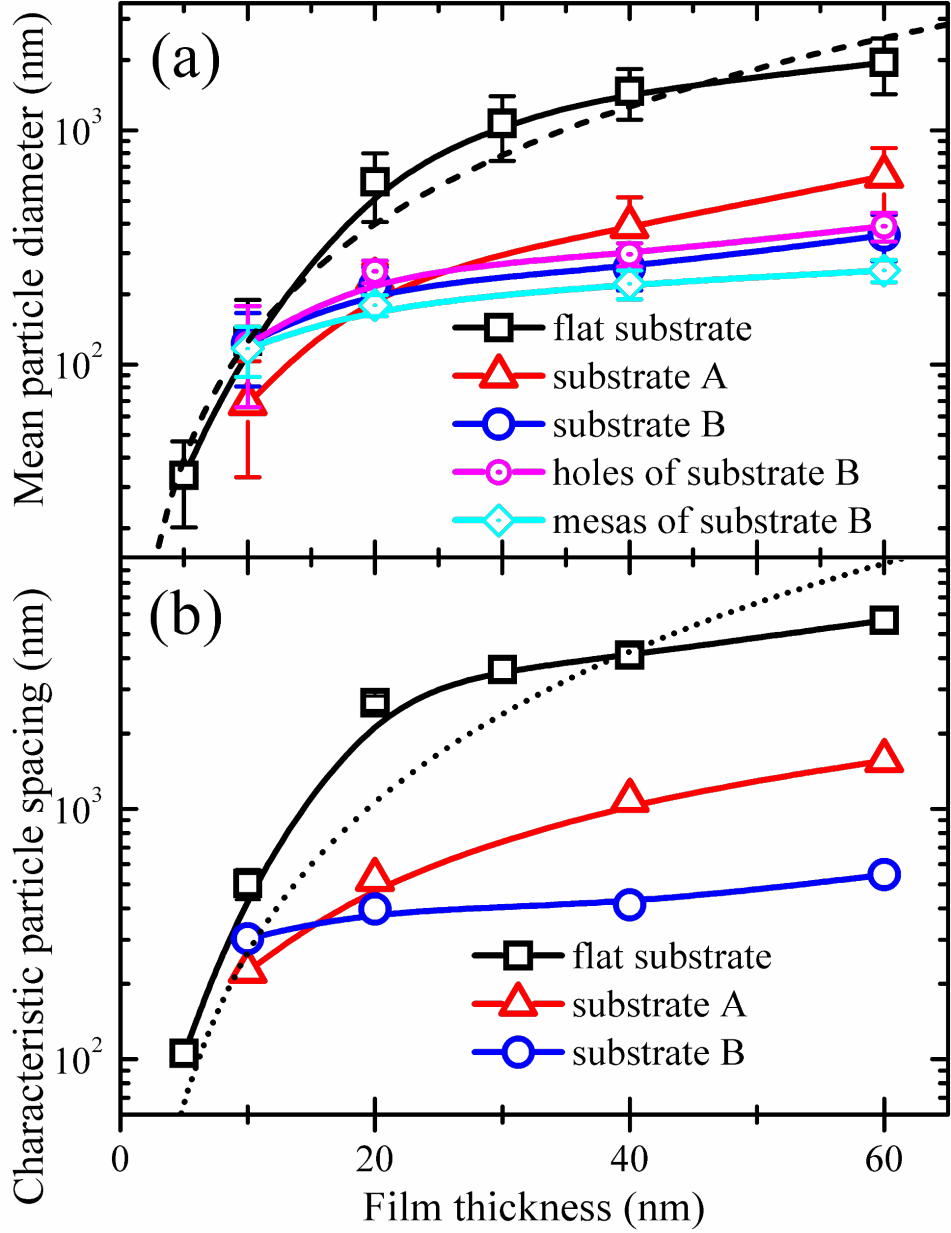
Plots of (a) the mean particle diameter and (b) the characteristic particle spacing as a function of the film thickness. The dashed line in (a) and the dotted line in (b) are the best fits for the flat substrate based on the variation of the mean particle size <*d*_p_> and characteristic particle spacing *s* with the film thickness *t* as <*d*_p_> 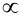 *t*^5/3^ and *s* 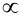 *t*^2^ [14,18,38].

Figure 7 displays the radially averaged autocorrelation function of Au particles dewetted from the 20 nm thick Au films on the different substrates. The radially averaged autocorrelation is calculated from the autocorrelation (also known as pair correlation) of pixels of a binary image as a function of their radial distance. The first minimum of this function gives the information on particle size, and the subsequent first maximum indicates the characteristic length (particle spacing). Comparing the plot of particles on the flat substrate, the plots of particles on both pre-patterned substrate A and B show a more periodic shape, indicating the high regularity of the nanoparticle arrays, which is also well confirmed by the autocorrelation images (insets in Figure 7). Characteristic particle spacing was determined based on the plan view SEM images and plotted as a function of the film thickness in Figure 8. Figure 8a shows the plot of mean particle size as a function of the film thickness. A similar trend is observed for the particles produced either on the flat substrate, or on the pre-patterned substrates, i.e., both mean particle size and characteristic particle spacing increase with increasing film thickness. In addition, both pre-patterned substrates A and B result in a clear reduction of the mean particle size and characteristic particle spacing as compared to the flat substrate. For film thickness below 20 nm, both mean particle size and characteristic particle spacing for the particles on substrate A are smaller than those for particles on substrate B. However, from 20 nm, on the contrary, both mean particle size and characteristic particle spacing for the particles on substrate A are larger than those for the particles on substrate B. It is also interesting to note that the characteristic particle spacing for the particles on substrate B first increases from 10 nm to 20 nm, then almost stays constant between 20 nm and 40 nm, and finally, increases from 40 nm to 60 nm. The characteristic particle spacing for the dewetted particles of the 20 nm and 40 nm thick Au films on substrate B (397 nm and 414 nm) should approximately equal the projected distance between the center of a hole and the center of the next mesa (363 nm), because there is typically only one particle in each hole and on each mesa. The characteristic particle spacing for the dewetted particles of the 20 nm thick Au film on substrate A and the 60 nm thick Au film on substrate B (522 nm and 546 nm) should equal the spatial period of the substrate structures (513 nm). However, there are deviations of these characteristic particle spacings within 10–40 nm, and this is probably due to the uncertainty of the radially averaged autocorrelation.

## Discussion

The presented results show that the characteristics of the dewetted particles (particle size and spacing) depend on the film thickness, and that the pre-patterned substrates lead to a reduction of particle size and spacing, and result in the formation of precise 2D particle arrays via dewetting. In addition, the substrate conformal imprint lithography (SCIL) technique enables the production of large areas of pre-patterned substrates with high uniformity and the corresponding fabrication of large areas of ordered 2D particle arrays. However, the respective influence of two substrate structures on the formation of particle arrays is different.

### Dewetting of the Au films on the flat substrate

According to the linear hydrodynamic spinodal dewetting theory for liquid films, the characteristic particle spacing *s* and the mean particle size <*d*_p_*>* vary with the film thickness *t* as *s* 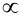 *t*^2^ and <*d*_p_*>* 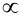 *t*^5/3^ [14,18,38]. Based on these variations, the best fit for the particle spacing *s* (dotted line in Figure 8b) doesn’t conform well to the experimental data, indicating that the solid-state dewetting of the polycrystalline films is different from the mentioned theory and may be more complicated, despite the good agreement between the best fit (dashed line in Figure 8a) and experimental data for the particle diameter <*d*_p_> on the flat substrate. Usually, the dewetting process of the solid films starts with void nucleation, and then proceeds with void growth and particle formation. For polycrystalline films on the flat substrates, void nucleation is generally thought to occur due to grain boundary grooving, via surface diffusion at the grain boundaries, and grain boundary triple junctions which intersect the substrate surface [39–40]. Recently, Mueller and Spolenak have reported that holes (large substrate-exposing voids) were found to protrude into the film predominantly at high angle grain boundaries during dewetting [41]. During annealing, grain boundary grooving and grain growth are competing kinetic processes. Grain growth is driven by the reduction of total grain boundary energy. The total grain boundary energy decreases with decreasing total grain boundary area. Microscopically, the reduction of the grain boundary area is accomplished by the movement of the individual boundaries and reducing the boundary curvature. To a first approximation, effective grooving will not occur during the rapid boundary moving [42], and even at high temperature, which facilitates surface diffusion, the effective grooving should be also retarded due to the upsetting of the rapid boundary movement. However, it has been shown that the velocity of boundary movement will decrease when the grain size reaches about three times the film thickness [43]. Subsequently, grooving occurs and in turn the formed grooves suppress the further boundary motion [40]. The mean diameter of the grown grains and the characteristic spacing of grain boundary triple junctions, which scale with the particle size and spacing for the dewetted particles in an approximate manner, increase with the film thickness. So it is expected that the nucleated void density decreases with increasing film thickness. In addition, an experimental investigation from Jiran and Thompson has shown that the void growth rate *R*_v_ decreases with increasing film thickness *t* dramatically (*R*_v_ 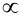 *t*^−3^) [32]. Therefore, it is reasonable that the particle size and particle spacing increases with film thickness (Figure 8).

#### Dewetting of the Au films on the substrate A (pyramidal pits)

**Figure 9:**
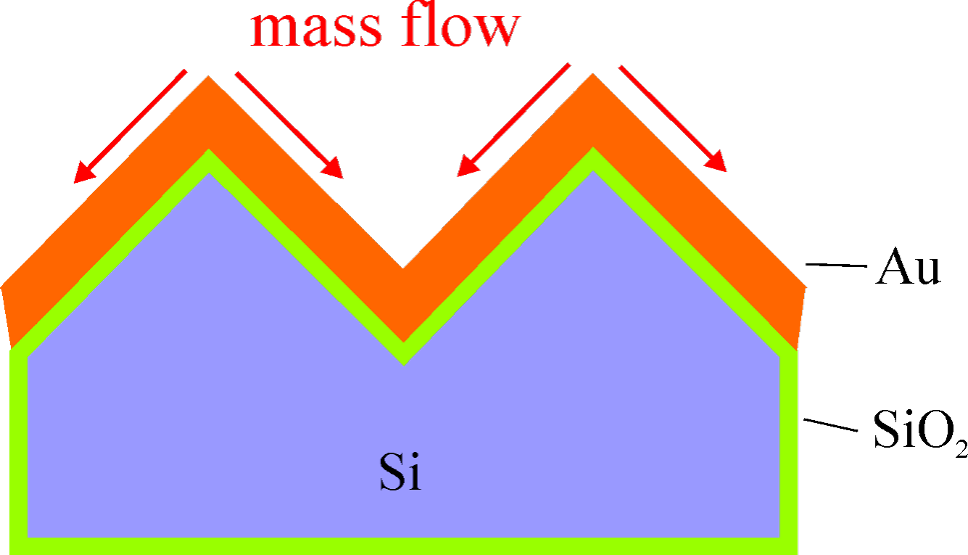
Schematic illustration of the mass flow induced by the local curvature driving diffusion on the substrate A.

Dewetting of the solid films is driven by the reduction of the surface energy (capillarity driven dewetting mechanism) and results in the formation of particles via surface diffusion. Surface curvatures of periodic substrate structures are associated with the chemical potential, introducing an additional driving force for the diffusion from the position with positive local curvature (peaks or ridges) to the position with negative local curvature (valleys), and an additional barrier for the diffusion crossing the position with positive local curvature (peaks or ridges) [9,11,25,35–36]. This is schematically shown in Figure 9. The occurrence of film thinning and subsequent film rupture or void nucleation at the peaks or ridges is expected due to the curvature driving diffusion, and the kinetics of this process depend on the film thickness for the structures with a fixed curvature. The formation of the precise particle array with one particle per pit on the substrate A (Figure 3b) confirms the influence of the periodic structure with curvature on the dewetting process. But the curvature driving diffusion is not the only process operating, and this process combined with the capillarity driven process and the grain growth leads to the formation of the precise particle array with one particle per pit only in the 20 nm thick Au film. However, the particle size and spacing are reduced clearly due to the modulation of periodic structure with curvature.

#### Dewetting of the Au films on the substrate B (circular holes)

Comparing to the substrate A, the substrate B has some different influences on the dewetting, resulting in the formation of the precise particle arrays with one particle per hole and/or per mesa in the 20 nm, 40 nm, and even 60 nm thick Au films. The interior sidewalls of the holes of the substrate B are perpendicular to the substrate surface, and the consequence is the evolution of the film discontinuity at the perpendicular interior sidewalls by the film deposition (Figure 5a and c). The film discontinuity is substrate-exposing, separates the film and limits the diffusion pathway within the individual regions (holes and mesas). The Au retracts from the discontinuity regions and one particle is formed in every individual region after the annealing, so that the precise particle arrays are evolved (Figure 4b–d). Figure 4b and c even shows the possibility to produce the precise 3D particle arrays by controlling the depth and areas of the holes and the area of the mesas.

**Figure 10:**
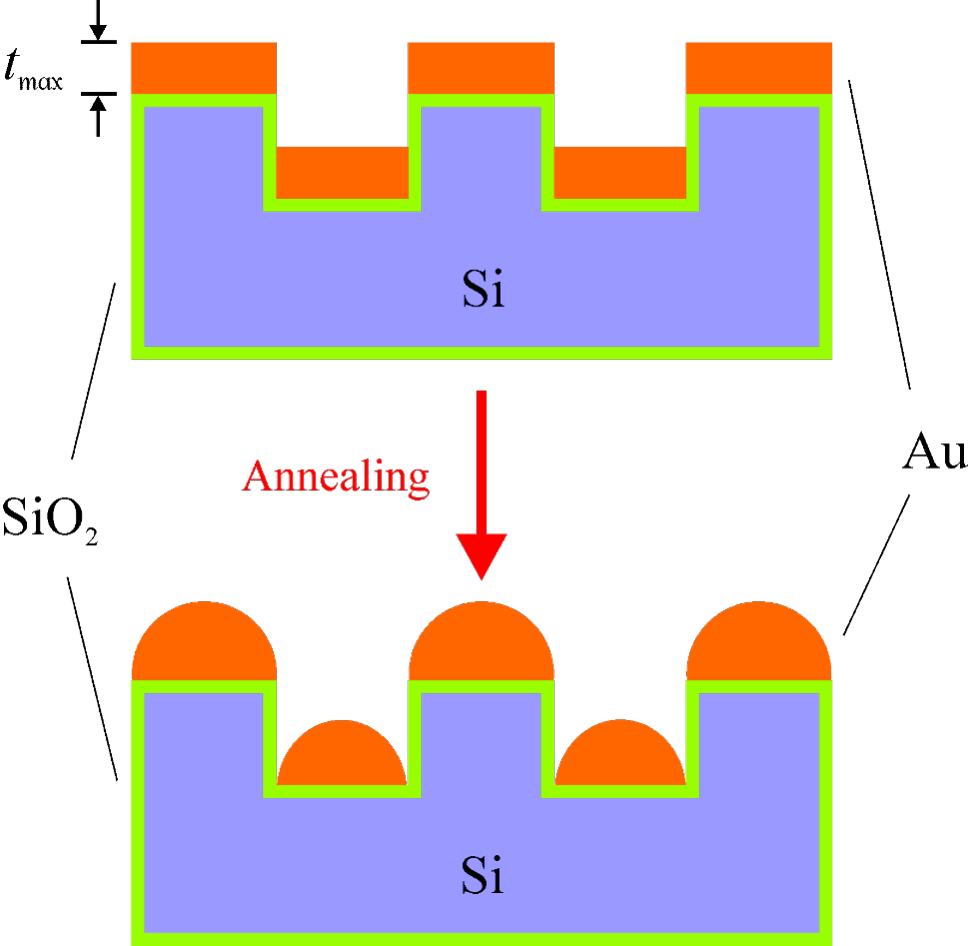
Schematic drawing of the particle formation on the substrate B via annealing induced dewetting.

Assuming that the particles are hemispherical, that the as-deposited films have a similar density to their bulk counterparts and that there is no material loss during annealing, the size of particle on the mesas can be calculated for the given as-deposited film thickness. Due to the structural constraint, the maximal size of the particles on the mesas is limited by the mesa dimension; otherwise the particles would be not stable on the mesas. Therefore, there is a corresponding maximal as-deposited film thickness (*t*_max_) for the stable particles on the mesas, as schematically presented in Figure 10. Consequently, the *t*_max_ for the mesas on the substrate B is calculated as about 45 nm. Altogether, for the 60 nm thick film on the substrate B, particles are prone to be formed in the holes which have a much larger area.

## Conclusion

We have studied the dewetting of Au films on the flat and pre-patterned substrates and discussed the influence of the substrate structures on the formation and characteristics of the dewetted particles. The pre-patterned substrates result in a clear reduction in particle size and particle spacing. Substrate A and substrate B have different influences on the dewetting, and both can lead to the formation of the precise 2D Au nanoparticle arrays. For that, the film thickness has to be adjusted to a structure-dependent value or thickness-window (around 20 nm for the substrate A, and ideally 20–40 nm for the substrate B). In addition, the possibility to fabricate precise 3D particle arrays is as well indicated by controlling the structure parameters of the substrate type B. The remarkable optical and plasmonic properties of the noble metallic particles indicate the potential applicability of this method in fabricating large areas of particle arrays for the plasmonic devices or in improving the efficiency of the photovoltaic devices and light-emitting diodes (LED) by modification of the surface optical properties.

## Experimental

The surface of (100) Si wafers was pre-patterned into a square array of pyramidal pits (substrate type A), shown in Figure 1a, and an array of circular holes with square symmetry (substrate type B), shown in Figure 1b, by employing the substrate conformal imprint lithography (SCIL) and reactive ion etching (RIE, Oxford Plasmalab 100 and STS 320 PC). The SCIL technique, which was developed by Philips Research and SUSS MicroTec, combines the advantages of both UV nanoimprint lithography techniques with rigid stamp for the best resolution and with soft stamp for the large-area (6 inch area) patterning. Thermal oxide several nanometers thick was grown on the pre-patterned Si to prevent reactions between the substrates and the subsequently deposited Au films. Additionally, 20 nm thick thermal oxides were also grown on a flat Si wafer for comparison. Au films with thicknesses *t* of 5 nm, 10 nm, 20 nm, 30 nm, 40 nm and 60 nm were deposited on the flat substrates and with thicknesses of 10 nm, 20 nm, 40 nm and 60 nm on the pre-patterned substrates using electron beam evaporation at a base pressure of 2 × 10^−7^ mbar. Film thicknesses were determined by the quartz crystal monitor and then verified by profilometer measurements (Dektak 150 – Veeco). After deposition, the films were annealed in pure N_2_ at 900 °C for 15 min in order to induce the dewetting. After a rapid initial heating from room temperature to 200 °C, it took 5 min for the further heating from 200 °C to 900 °C. Dewetted particles were investigated using ultra-high resolution scanning electron microscopy (SEM, Hitachi S-4800). Particle sizes were calculated as circular diameters and determined using a threshold image contrast in the SEM images and performing a pixel count.
